# Purpura induced by laser hair removal: a case report

**DOI:** 10.1186/s13256-018-1604-4

**Published:** 2018-02-27

**Authors:** Abdullah Al-Hargan, Yasser A. Ghobara, Ahmad Al-Issa

**Affiliations:** 1 Dermatology Department, Prince Sultan Military Medical City, Riyadh, Saudi Arabia; 2 Derma Clinic, Riyadh, Saudi Arabia

**Keywords:** Laser hair removal, Alexandrite laser, Side effects, Purpura, Case report

## Abstract

**Background:**

Laser hair removal is an effective and safe method for the permanent reduction of unwanted hair. Common side effects include temporary pain, transient erythema, and perifollicular edema. Purpuric eruption is a rare adverse event.

**Case presentation:**

To the best of our knowledge, this is the second case report of purpura induced by laser hair removal. Our patient is a 50-year-old woman of Arab origin. Her positive reaction to a laser hair removal provocation test helped in the diagnosis; her condition was managed with an orally administered corticosteroid, leading to complete resolution within 5 days.

**Conclusion:**

Purpura induced by laser hair removal is a self-limiting and unusual side effect; physicians’ awareness of such adverse events can help them to avoid unnecessary investigations and provide guidance for better management.

## Background

Laser hair removal (LHR) has become a popular modality for the removal of unwanted hair; it is typically performed or supervised by laser-trained dermatologists, and it is the most requested cosmetic procedure in the world [1]. LHR is not a standardized procedure, as the parameters are individualized, so it requires accurate laser selection, optimal pulse duration, and appropriate fluence to achieve highest efficacy and safety [2]. Therefore, background knowledge regarding the practical application of laser-tissue interactions and the principles of selective photothermolysis are vital for safe LHR practice [3]. In general, LHR is a safe modality, and common side effects include temporary pain, transient erythema, and perifollicular edema; these adverse events depend on variable factors such as skin type, treatment site, laser system, parameter set, and operator knowledge [4]. Unfortunately, untrained or unsupervised non-physician providers can practice LHR, increasing the risk of avoidable complications [5]. Here we present the second case report in the literature that highlights the self-limited nature of LHR-induced purpura, and we describe how similar cases should be handled.

## Case presentation

**Fig. 1:**
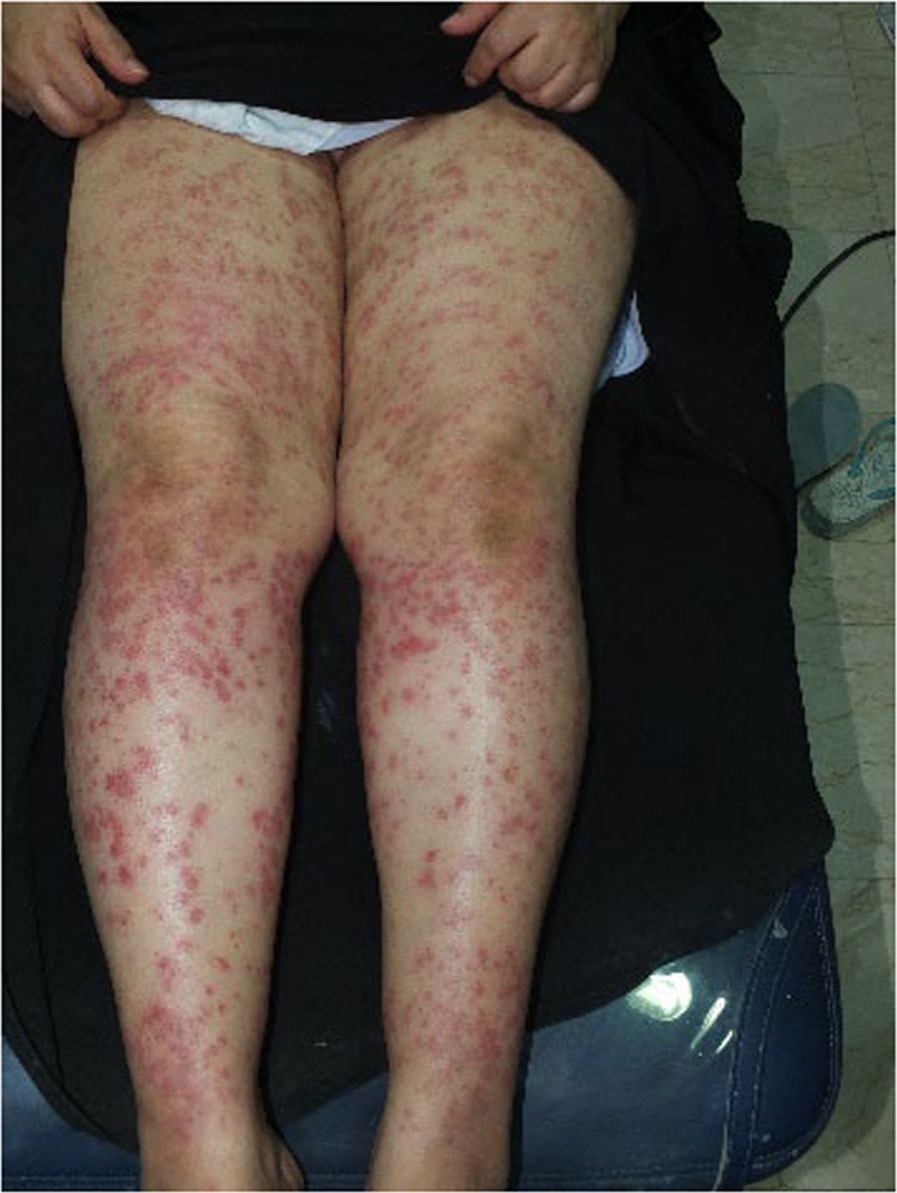
Numerous palpable purpura over both thighs and legs after laser hair removal

A 50-year-old woman of Arab origin (with skin phototype III) came to our clinic presenting with a mildly itchy skin eruption over her bilateral lower extremities (Fig. 1) 2 days after undergoing a long-pulsed 755-nm alexandrite LHR procedure (GentleLASE; Candela Corporation, Wayland, MA, USA).

A clinical examination showed multiple round, non-blanching erythematous papules on both of her thighs and legs. She was vitally stable and had no systemic symptoms. She had no history of underlying systemic disease and no history of recent infection; she was not taking any medications. She also had no history of similar eruptions from other laser treatments. Basic laboratory screenings (complete blood count, prothrombin time, partial thromboplastin time, and international normalized ratio) were unremarkable, and workups for connective tissue diseases, cryoglobulinemia, and infectious etiologies were all negative. She declined a skin biopsy but agreed to less invasive provocation testing (Table 1) on her uninvolved right forearm at a lower parameter setting.

**Table 1:** Provocation test

Test	Alex 755 nm + DCD	Alex 755 nm – DCD	ND:YAG 1064 nm + cold air	DCD only	LN
Spot size (mm)	18	18	15	NA	NA
Pulse duration (ms)	3	3	15	NA	NA
Fluence (J/cm^2^)	8	8	20	NA	NA
Frequency (Hz)	1.5	1.5	1.5	NA	NA
Reaction	+	+	++	–	–

*Alex* alexandrite, *DCD* dynamic cooling device, *LN* liquid nitrogen, *NA* not applicable, *ND:YAG* neodymium-doped yttrium aluminum garnet

**Fig. 2:**
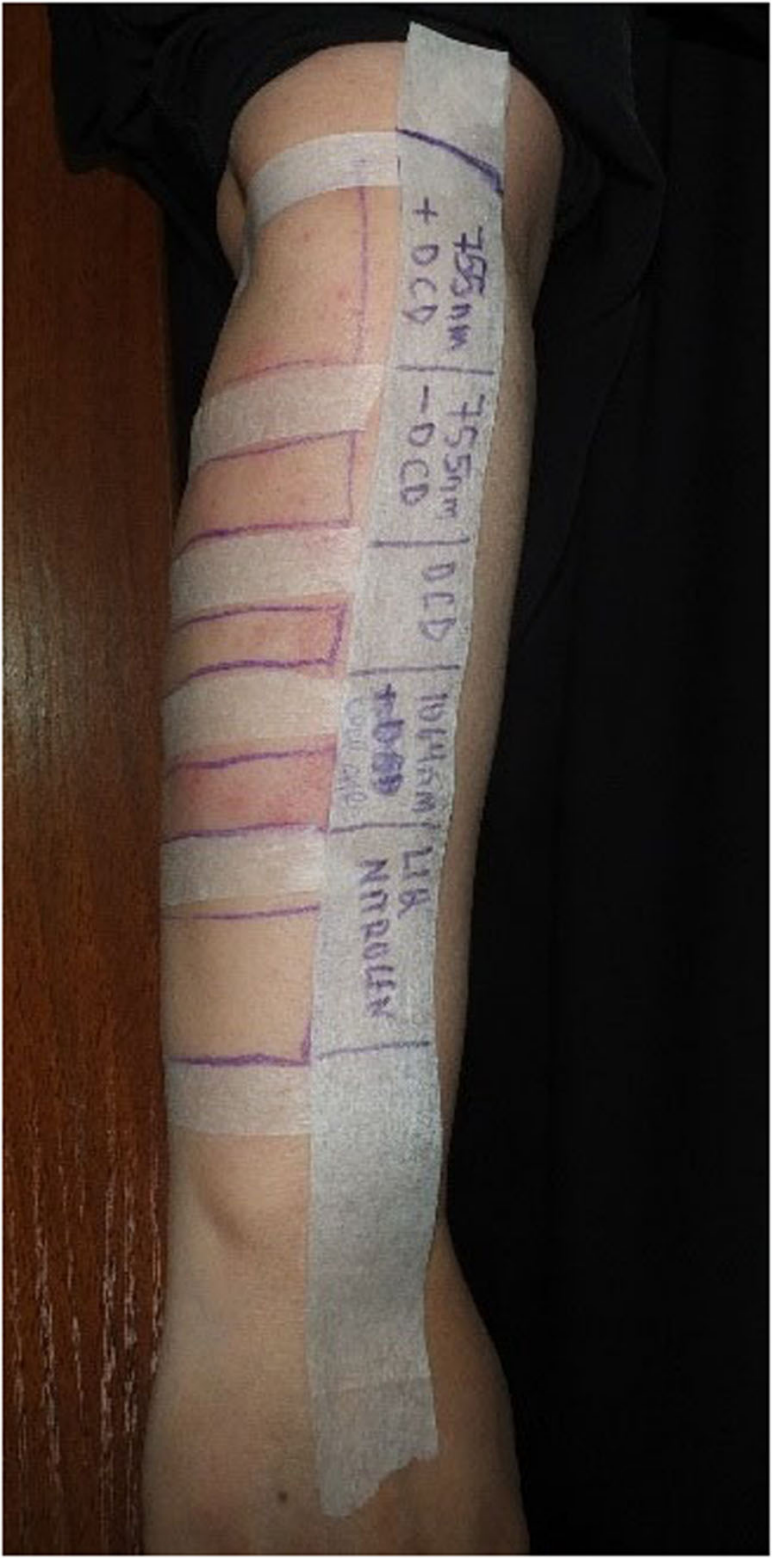
Provocation test of right dorsal forearm. Long-pulsed alexandrite and neodymium-doped yttrium aluminum garnet shows positive reaction 24 hours after test spot

This test revealed: a mild positive reaction to a long-pulsed 755-nm alexandrite laser; a severe positive reaction to a long-pulsed 1064-nm neodymium-doped yttrium aluminum garnet (Nd:YAG); and negative reactions to tests involving ice, cryogen (1,1,1,2 tetrafluoroethane), and liquid nitrogen (LN). Taking into consideration the positive reaction to LHR devices (Fig. 2), a diagnosis of LHR-induced purpura was made. Our patient was prescribed orally administered prednisone (0.5 mg/kg per day). At a follow up 5 days later, she showed complete resolution of her symptoms.

## Discussion

LHR is an effective and well-tolerated method for the long-term reduction of unwanted hair growth. Most side effects are transient and resolve spontaneously; however, it may cause rare but serious complications when used improperly by untrained personnel [6]. All LHR systems target follicular melanin and therefore provide a significant chance of epidermal or dermal injury during the epilation process [7]. LHR-induced purpura is an unusual side effect occurring in 7% of cases using LHR devices; it is more common for patients with darker skin types and for treatments on the extremities [7]. A case similar to ours was reported in the literature; in this case, a middle-aged woman with skin type II developed palpable purpura after undergoing hair removal with an alexandrite laser, but the condition resolved completely with only conservative treatment within 6 weeks [8]. Although skin biopsy is a helpful diagnostic method, patients may refuse or decline such an invasive procedure; therefore, a less invasive technique such as a provocation test may be appropriate to help in the diagnosis [9, 10]. We suggest reassuring the patient and providing a short course of orally administered systemic corticosteroid therapy to accelerate clinical resolution.

## Conclusions

LHR-induced purpura is a rare and self-limited adverse event, and physicians’ awareness of such side effects can help them to avoid unnecessary investigations and provide guidance for better management. Further studies are required to understand the pathogenesis of this condition, and additional case reports will help in recognizing the condition’s risk factors, patient characteristics, and environmental influences.

## Abbreviations

LHR: Laser hair removal
LN: Liquid nitrogen
Nd:YAG: Neodymium-doped yttrium aluminum garnet
